# The Influence of Social Media Interactions and Behaviors on Depressive Symptoms Among Sexual and Gender Minority Young Adults in the United States: Protocol for a Mixed Methods Longitudinal Study

**DOI:** 10.2196/43627

**Published:** 2023-01-24

**Authors:** César Escobar-Viera, Robert W S Coulter, M Reuel Friedman, Brian Thoma, Galen E Switzer, Jamie Martina, James Erin Egan, Brian Primack

**Affiliations:** 1 Department of Psychiatry School of Medicine University of Pittsburgh Pittsburgh, PA United States; 2 Department of Behavioral and Community Health Sciences School of Public Health University of Pittsburgh Pittsburgh, PA United States; 3 Department of Urban-Global Public Health School of Public Health Rutgers University Piscataway, NJ United States; 4 Division of General Internal Medicine School of Medicine University of Pittsburgh Pittsburgh, PA United States; 5 Department of Psychiatry School of Medicine University of Pittburgh Medical Center Pittsburgh, PA United States; 6 Department of Health Promotion and Health Behavior College of Public Health and Human Sciences Oregon State University Corvalis, OR United States

**Keywords:** mixed methods, longitudinal, depression, sexual and gender minorities, social media

## Abstract

**Background:**

Sexual and gender minority (SGM; ie, lesbian, gay, bisexual, transgender, and otherwise queer) young adults experience disparities in depression and other internalizing psychopathology. Although social media use is widespread and SGM people have more social media accounts and are more socially active on them than non-SGM individuals, few studies have examined the impact of social media on depression in this group.

**Objective:**

The PRIDE iM study will be the first longitudinal, mixed methods research conducted to determine the impact of social media interactions and behaviors as pathways to depressive symptoms among SGM young adults living in the United States.

**Methods:**

PRIDE iM uses a *bookends variation* of the longitudinal sequential mixed methods design. Participants will be recruited nationally from social media. First, between July 2019 and February 2020, we conducted a qualitative phase (T1) comprising web-based individual interviews (N=58) to inform the building and content of the quantitative survey. Second, from February 2022 to September 2022, we will conduct a series of web-based surveys (N=1000 at baseline) with 4 data points (T2-T5), each one collected every 6 to 8 weeks. Third, from October 2022 to December 2022, we will conduct a second qualitative phase (T6) of web-based interviews using outcome trajectories found in the longitudinal survey analyses to purposively sample survey participants and conduct web-based interviews to contextualize and explain survey findings. Qualitative data from T1 and T6 will be analyzed using a reflexive thematic analysis approach. As we sought to capture change over time in the association between the main predictors (ie, social media interactions and behaviors) and depressive symptoms, we propose analyzing T2 to T5 data using latent growth models with a structural equation modeling framework. Data integration at the method, interpretation, and reporting levels will be achieved through building and connecting and the use of a staged approach and joint displays, respectively. At all stages, we will assess the fit of data integration as recommended by the principles of best practice for mixed methods research in psychology.

**Results:**

Data collection will be completed by December 2022. Qualitative data analyses will be completed by March 2023, and quantitative analyses of the primary outcome of interest will be completed by June 2023.

**Conclusions:**

PRIDE iM will confirm, reject, or uncover the presence of potential relationships between social media interactions and behaviors and depressive symptoms among SGM people. This study represents fundamental groundwork to develop social media–based interventions that target modifiable interactions and behaviors that are most likely to influence mental health outcomes, thus seizing the opportunity to merge the popularity of this medium among SGM people with evidence-based approaches.

**International Registered Report Identifier (IRRID):**

DERR1-10.2196/43627

## Introduction

### Background

Sexual and gender minority (SGM; ie, lesbian, gay, bisexual, transgender, and otherwise queer [LGBTQ+]) young adults experience well-documented disparities in both internalizing [1,2] and externalizing psychopathology [3] compared with their cisgender heterosexual peers. These mental health disparities persist into adulthood and are present even among SGM people who identify as such late in their adolescence or young adulthood [4]. Minority stress theory [5] poses that, given the dominant culture, norms, and social structure, SGM people are likely subjects of an incongruence between information provided by society on how the world works and the minority person’s experience of the world, determining stress processes that may worsen depression. Minority stress is the prevalent framework used to explain disparities seen in depression among SGM people; expansions of it have been developed for specific conditions such as substance use [6], cardiovascular disease [7], suicidal thoughts and behavior [1] and certain SGM groups such as adolescents [8,9] and transgender persons [10-13].

Social media encompasses a variety of websites and mobile apps (eg, TikTok, Snapchat, and WhatsApp) that enable users to create and share content and participate in web-based social networking [14]. Over 90% of adults in the United States have at least one social media account, which they use for an average of 2 to 4 hours daily, with YouTube, Facebook, and Instagram among the most popular platforms, and people aged 18 to 30 years being the group with the most sustained use growth among adults [15]. In addition, the affordances of social media (eg, asynchronicity, absence of physical cues, permanence of interactions, quantifiability via likes, and upvotes) have deeply changed the way in which people socialize, connect with others, build relationships, and seek and receive support and information [16,17].

On average, SGM people are more socially active on social media and more likely to have multiple social media accounts than non-SGM individuals [18-20]. Moreover, SGM persons seek and engage with more web-based support chats than their non-SGM peers [21]; seek web-based support resources to help them navigate challenging settings where both racial and sexual minority stigma might be salient [22,23]; and use social media to connect with others like them, increase their sense of belonging, and increase emotional well-being [24]. Despite these positive experiences, negative social media interactions also give way to social comparison, stigmatization, cybervictimization, and other forms of discrimination, which in turn might influence depressive symptoms [25,26] and other psychopathology [27,28].

### Current Research Gaps

**Figure 1:**
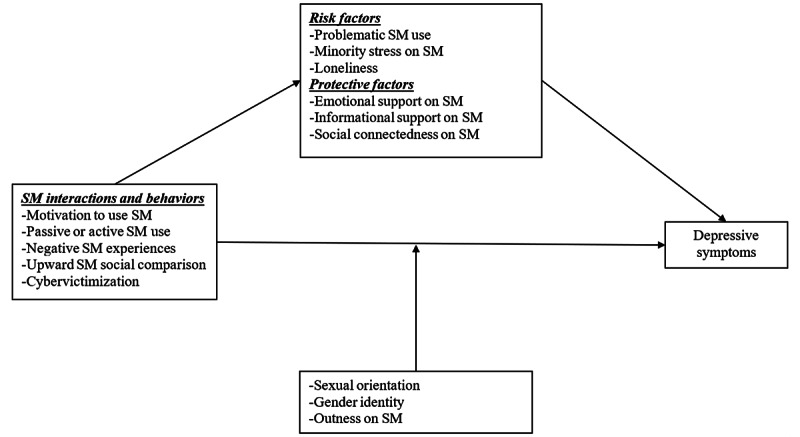
Model depicting risk and protective factors in the pathways of characteristics of social media interactions and behaviors and depressive symptoms among sexual and gender minority young adults. SM: social media.

Although there is qualitative evidence available from SGM people indicating the salience of social media interactions to them [23,26], few studies have been conducted to determine the impact of social media interactions on mental health outcomes among SGM individuals, mostly among college students [29]. Moreover, the available studies follow the recent literature trend of focusing on determining the effect of the amount of time spent on social media (eg, time elapsed while using social media, number of checks per unit of time, and number of platforms used) on mental health. In the general population, the former approach showed mixed findings of positive [30,31], bidirectional [32], or no association [33] between quantity of social media use and mental health outcomes over time. These conflicting findings are indicators that we need a new approach that focuses on studying the factors that matter to SGM social media users, including the types of interactions and behaviors on social media platforms and their impacts on mental health. This is relevant as it is during these experiences on social media that SGM persons might encounter some of the minority stressors or protective factors [5,8]. On the basis of minority stress and our preliminary work [25], we propose a mediation model (Figure 1) in which the relationship between specific social media interactions and behaviors and depressive symptoms is mediated by both protective and risk factors (eg, emotional support experienced on social media). An additional issue that arises is that quantitative results alone are inadequate to describe and fully explain SGM young adults’ social media behaviors and interactions or their effect on their mental health. Therefore, a mixed methods approach has the potential to unveil stressors and protective factors uniquely shaped by the affordances of social media, which may influence mental health outcomes in ways that could be different from similar offline interactions and behaviors.

### Study Objectives

The PRIDE iM study will be the first longitudinal, mixed methods research conducted to determine the impact of several forms of social media interaction and behavior on the pathways of depression and other mental health outcomes among SGM young adults living in the United States (Multimedia Appendix 1). To address critical methodological factors needed to ensure a high-quality longitudinal study with SGM people, the main characteristics of PRIDE iM include (1) recruitment via social media to reach members of an often-marginalized group who are users of the technology under study, (2) recruitment of young adults aged 18 to 30 years as this age group recently experienced the most sustained growth in social media users, (3) presurvey qualitative assessments to identify relevant SGM-specific social media interactions and behaviors and add them to the survey, (4) repeated measures of potentially critical risk and protective interactions and behaviors on and off social media, (5) postsurvey qualitative assessments with survey participants to contextualize the trajectory of depressive symptoms, and (6) a mixing analysis phase to identify similarities and differences across groups. Upon completion, the PRIDE iM study will provide critical data in terms of intervention targets, such as reducing the occurrence or impact of risk factors (eg, upward social comparison) or boosting protective factors (eg, social connectedness) for depression and other negative mental health outcomes among SGM people—a high-need, underserved, and hardly reached population.

## Methods

### Study Design

**Figure 2:**
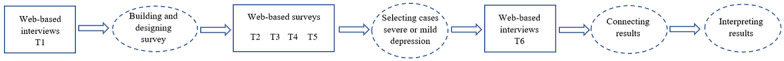
PRIDE iM study design. T: time.

Given the longitudinal nature of our study, we leveraged a multistage mixed methods framework in which we combined both exploratory and explanatory approaches [34]. PRIDE iM uses a *bookends variation* of the longitudinal sequential mixed methods design [35], which is a combination of prospective and retrospective studies. In this design, we gather qualitative data in the form of individual interviews at both the beginning (T1) and the end (T6) of the study and collect quantitative data in the form of 4 longitudinal web-based surveys (T2, T3, T4, and T5) at nonconcurring times with the collection of qualitative data. Figure 2 provides an overview of the study design, including timing of data points for both quantitative and qualitative data strains as well as for analyses, data mixing, and interpretation of results. Throughout this paper, we use T1 to T6 to designate each point of data collection.

The design that we chose was appropriate for 3 reasons. First, we used a qualitative phase (T1) comprising web-based individual interviews to inform the building and content of the quantitative survey. Although the existing literature provided a list of potentially relevant risk and protective forms of social media interactions and behaviors for depression among the general population [36,37], we focused on social media experiences involving minority stress that are unique to SGM persons and that might help explain depressive symptoms among this group. We decided to conduct individual interviews, and this was appropriate to balance a need for an in-depth understanding of the impact of social media experiences on mental health with the need to protect individuals’ privacy and sensitive information. Second, we used a longitudinal web-based survey study with 4 data points (T2-T5), each one collected every 6 to 8 weeks. We sought to capture change in the association between the main predictors (ie, social media interactions and behaviors) and outcome variables (ie, depressive symptoms) over time. Using these 4 data points, our analyses will be able to identify trajectories of depressive symptoms over time. Third, given the limitation of quantitative data to fully explain the change in social phenomena over time, we will conduct a second qualitative phase of web-based interviews (T6). We will use the outcome trajectories identified in the T2 to T5 quantitative analyses and form groups based on these trajectories for conducting web-based interviews with SGM persons who participated in the survey in each of these groups. We will use the interviews to contextualize trajectories found in survey analyses and then conduct a comparative analysis of qualitative findings by groups identified in the survey (eg, respondents with severe vs mild depressive symptoms) to describe similarities and differences between said groups.

### Ethics Approval

All recruitment and data collection procedures were approved by the University of Pittsburgh Institutional Review Board (study 19050007; approval June 20, 2019). We sought and obtained informed consent from potential participants before completing any of the research activities (web-based interviews or web-based surveys) of the study. Given that PRIDE iM study participants can be located anywhere in the United States and as the study posed minimal risk for participants, we obtained a waiver for documenting consent. We included all the standard components in the web-based informed consent forms, including study goals and description and estimated time required to complete the activity. Participants receive web-based gift cards in compensation for their time taking part in each of the research activities. We collected no identifiable information other than participant email addresses, phone numbers, and IP addresses (which were collected automatically by the web-based survey platform). This was necessary to schedule participation in the web-based interviews and to send gift cards to the participants after completing a research activity. Given the inclusion of sensitive topics and questions in both the interview and survey, we inform participants that they can choose to stop taking part at any time if they feel uncomfortable. In the web-based surveys, if the participant answers affirmatively to any of the suicide-related questions, a pop-up screen appears where we provide the contact information of nationally available resources for both SGM people and persons with suicidal ideation. These same resources appear listed at the end of the survey, and we provide the name and contact information of the principal investigator for more personalized resources.

### Recruitment and Data Collection Procedures

#### Sample Selection

We used a purposive sampling approach throughout the 3 phases of the PRIDE iM study. This was appropriate as purposeful sampling permits identifying and selecting study participants who experience firsthand the phenomenon under study, making this approach ideal for mixed methods research studies [38]. To recruit participants, we used targeted advertisements on social media, which have been previously used with success for the recruitment of SGM people to both cross-sectional and cohort studies [39,40]. This was appropriate for multiple reasons. As noted previously, SGM people are heavy social media users, and social media recruitment facilitates the enrollment of a diverse sample of SGM individuals, including those who have not disclosed their sexual orientation or gender identity, those who live in rural areas, and those who do not participate in community-based organizations that serve SGM persons [41]. Throughout the 3 phases of the study, individuals were eligible if they were aged 18 to 30 years; resided in the United States (as determined by zip codes); identified as LGBTQ+; and had at least one social media account and used it on at least a weekly basis.

#### First Series of Web-Based Interviews (T1)

##### Recruitment

The recruitment approach described previously was appropriate for the purpose of these interviews, which was to inform constructs and items for survey development through learning from the lived experience of SGM young adults who use social media and whether and how this use affected their mental health and well-being. In this way, the qualitative data strain served the purpose of integration through *building*, in which the results of one data collection and analysis activity inform the data collection of the other research activity [34]. Recruitment took place between July 2019 and February 2020. To create our advertisements, we used the advertisement creation feature on Meta Ads Manager (Meta Platforms, Inc; which allows for cross-posting to Facebook and Instagram) and set advertisements to be shown solely to people living in the United States. At the time, the Facebook advertisement manager allowed for targeting specific audiences based on location, age, interests (including “Interest tags” specific to the LGBTQ+ community and LGBTQ+ topics), and activity. After seeing the advertisements, interested individuals clicked on “Learn more” and were redirected to a study website that provided more information about the study goals, expected involvement, information about compensation, and an anonymous link to an eligibility questionnaire housed on the Qualtrics survey platform (Qualtrics International, Inc). Eligible individuals were invited to provide their preferred method of contact (ie, phone number or email address) so that a research assistant could contact them to schedule a time and date for the interview.

A total of 72 hours before the web-based interview, potential participants were sent an email with a link to the web-based informed consent form for their preliminary review. We encouraged potential participants to read the informed consent form and reply to the email with any clarification questions they might have. The day before the interview, we sent a final email with the link to the web-based conference platform where the interview would take place. At the date and time agreed upon for the interview, potential participants clicked on the link provided; this link took them to the formal informed consent form, and after providing consent, participants were admitted to the web-based conference room.

##### Data Collection

The web-based interviews were 60 minutes long. We used a Health Insurance Portability and Accountability Act–compliant platform [42]. To protect the individuals’ privacy, only audio was recorded during each interview. The interviews explored motivations for using social media and perspectives on both positive and negative social media interactions as well as the perceived impact on mental health and well-being. After completing the interview, participants received US $50 gift cards in compensation for their time. The recordings were transcribed verbatim using a professional web-based service.

#### Web-Based Longitudinal Survey (T2-T5)

##### Recruitment and Initial Screening

Recruitment for the baseline assessment (T2) took place from February 2022 to March 2022 via targeted advertisements through Meta Ads Manager. However, as of January 2022, Meta updated its new audience targeting policy to protect the privacy of vulnerable populations [43]. The new policy eliminated the option of specifically targeting interests related to LGBTQ+ identities as well as religious or political interests. We chose an alternative option that consisted of creating a “custom audience” within the Meta Ads Manager tracking system to target future advertisements to a “lookalike audience.” After publishing a set of advertisements, a custom audience is created using the first 1000 to 5000 users that click or tap on the ads. This custom audience is then used so that ads can be seen by people who previously interacted with content on Instagram and Facebook [44]. A “lookalike audience” comprises users who are similar to the custom audience that first interacted with the advertisement published on Instagram and Facebook [45]. Once this custom audience is created, future iterations of the advertisements are shown to new people who share similar characteristics (ie, “lookalike”) with the custom audience.

**Figure 3:**
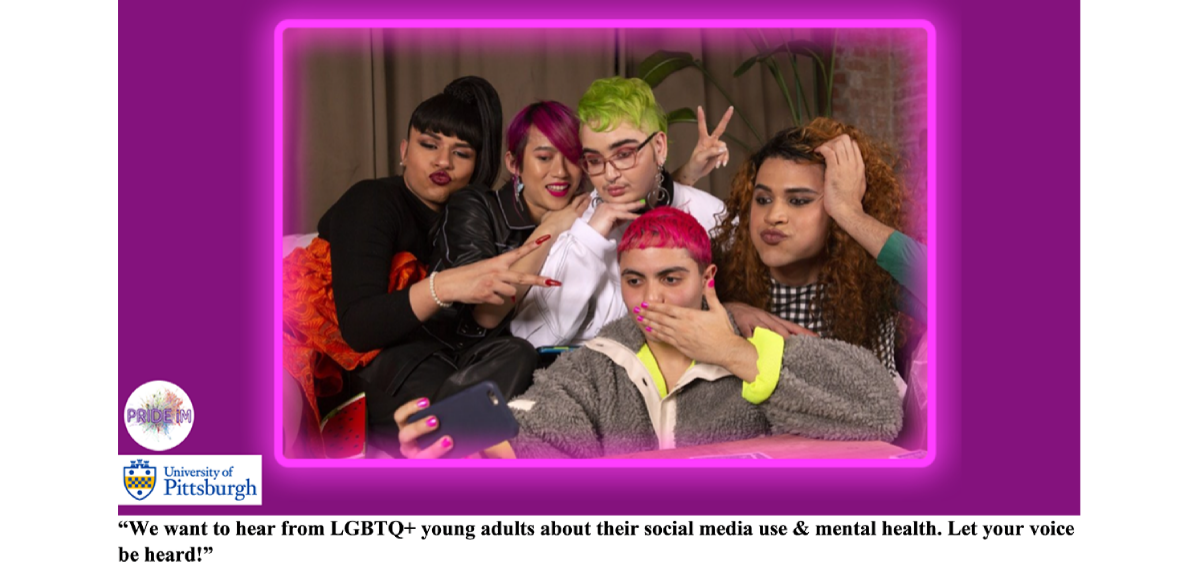
Example of one of the PRIDE iM social media recruitment advertisements. LGBTQ+: lesbian, gay, bisexual, transgender, and queer.

In total, 7 copyright-free images that would appeal to a broad base of SGM persons were purchased from iStock by Getty Images for web-based advertisements. Images depicted same-sex or queer-gendered couples, a group of smiling young adults of various backgrounds and hairstyles, and a rainbow-colored heart light-emitting diode light without any humans. Figure 3 shows an example of one of the advertisements published on social media. After clicking on the advertisement, persons were directed to an institutional study website where they could find more detailed information that was beyond the character limit allowed on social media advertisements, including the goals of the study, eligibility criteria, expected commitment (ie, completing four 30-minute surveys over 7-8 months), and participant compensation. Those who remained interested were able to click on a link to an eligibility screening questionnaire housed on the Qualtrics survey platform. If eligible, potential participants were invited to provide informed consent so that they could gain immediate access to the full web-based baseline questionnaire.

After completing the baseline survey, participants were asked to provide their email address for payment, their phone number, and their preference for a contact methodology (eg, email, phone call, or SMS text message). Email addresses were used to send web-based gift cards to all participants who completed at least half of the baseline questionnaire as well as reminders for follow-up surveys.

##### Participant Retention Strategies for Follow-up Surveys (T3-T5)

Given both the web-based nature and the longitudinal design of this study phase, reducing participant attrition was a major concern. A recent meta-analysis found that using a mix of retention strategies delivered via the internet improved participant retention rates in longitudinal cohort studies [46]. First, we sought to reduce barriers to participation by prioritizing survey measures hypothesized to have an association with the primary outcome of interest (ie, depression) according to the existing literature and our conceptual model (Figure 1) and allowing for extended data collection windows (up to 1 week) for each wave. Second, to build a community around the study and our research program, we created a study brand (PRIDE iM study) and a logo, website, and social media presence on 3 social media platforms (Twitter, Instagram, and Facebook). Social media accounts and websites were created 6 to 12 months before the formal study started and were used to update our followers about ongoing research conducted by our team and others on the topic of use of communication technologies and LGBTQ+ mental health. Third, we used a variety of reminders and follow-up strategies, including bimonthly e-newsletters (sent using Mailchimp [Rocket Science Group], a web-based platform and email service that allows for managing mailing lists) in between periods of data collection, email reminders sent 72 hours before the next follow-up survey, SMS text message reminders (only in cases of participants who authorized being sent SMS text messages), and incentives of increasing value over time (gift cards of US $10 for T2, US $15 for T3, US $20 for T4, and US $25 for T5). Finally, toward the end of each data collection period, we sent targeted emails or SMS text message reminders (according to each person’s preference) to participants whose responses we still had not received.

##### Eligibility Determination for Follow-up Surveys (T3-T5)

Preventing and detecting fraud in web-based survey studies with participants recruited from social media is an ongoing challenge among researchers [47]. Fraudulent surveys include duplicate responses from the same participant using different email addresses, providing response patterns, or reaccessing the survey with false responses after being deemed ineligible in the first attempt. To minimize these risks, we implemented a series of combined red flags based on previous literature [39,40,48,49] and our own team’s experience. Once data collection for the baseline (T2) survey was completed, we downloaded all the data from Qualtrics. A team of research assistants not involved in data analysis or interpretation created variables representing aspects related to survey participation that could be useful to identify fraudulent responses, such as geographic location (including approximate latitude and longitude), IP address, start and end date and time, survey completion and time duration for completion, attention-control questions [50], phone number, and email address. We used 3 criteria to determine fraudulent responses (Textbox 1).

**Criteria to determine fraudulent survey responses.**

1. Location outside the United States according to *one* of the following:
  Location data from Mailchimp (Rocket Science Group)
  Latitude and longitude data from Qualtrics (Qualtrics International, Inc)
2. More than 1 response per IP address:
  If only 2 responses, keep both if they did not meet criterion 1
  If >2 responses, keep the first 2 if none of the responses met the previous criterion 1; if any of the responses meet criterion 1, remove all of them
  Responses that started or ended <3 minutes apart (in these cases, only the first response was retained as part of the data)
3. More than 1 response per either email address or phone number (in these cases, only the first response was retained as part of the data)

Responses that met any of the criteria listed in Textbox 1 were removed from the baseline data; those participants did not receive compensation and were no longer contacted for the follow-up surveys. In addition, some baseline participants completed the survey but provided low-quality data, either because their completion time was <6 minutes or because they completed less than two-thirds of the survey. In these cases, participants were compensated for the baseline survey but were no longer contacted for follow-up surveys, and their responses were removed from the data.

##### Data Collection for Follow-up Surveys (T3-T5)

Eligible participants were sent email invitations and reminders for T3 to T5 surveys starting in early April 2022 (1 week after we completed baseline data collection). Follow-up surveys were hosted on Qualtrics, and responses were collected for 30 days during May 2022 (T3), July 2022 (T4), and September 2022 (T5). On the first day of each month, we sent an email to each participant with a unique link to the survey. To protect individuals’ right to timely information about the study, we sought reconsent from all participants before allowing access to every follow-up survey. To maintain rapport and participants’ interest in the study, we sent 1 e-newsletter during the month in between data collection (mid-April, mid-June, and mid-August) and 1 reminder 3 days before sending the survey links. Finally, we sent target emails or SMS text message reminders (depending on their preference for receiving direct communication) to the participants who had not yet completed the follow-up survey 1 week before the end of each data collection period.

##### Survey Measures

The baseline (T2) and follow-up (T3-T5) surveys incorporated different sections in which we administered the measures outlined in Textbox 2 based on our conceptual model (Figure 1).

**Measures administered during the baseline (T2) and follow-up (T3-T5) surveys.**

**Primary outcome of the study**

- We seek to determine changes in depressive symptoms, which have been consistently described in the literature as disproportionately more prevalent among sexual and gender minority (SGM) individuals. *Depressive symptoms were assessed* using the 9-item Patient Health Questionnaire [51]. In addition, we assessed other mental health problems that have been consistently found to be more prevalent among SGM people, including *generalized anxiety disorder symptoms*, measured using the Generalized Anxiety Disorder-7 [52]; *sleep disturbance symptoms*, measured using the 4-item Patient-Reported Outcomes Measurement Information System (PROMIS) [53]; *suicide ideation in the past month*, measured using a subscale of the Columbia Suicide Severity Rating Scale [54]; *eating disorder symptoms*, measured using the Sick, Control, One, Fat, Food screening questionnaire [55,56]; *cigarette smoking*, measured using 2 modified items from the Behavioral Risk Factor Surveillance System questionnaire [57]; *alcohol use in past month*, measured using a modified version of a 1-item screener by Smith et al [58]; and *marijuana use in the past month*, measured using an item modified from the Youth Risk Behavior Survey [59].

**Primary predictor**

- Given that the focal interest of this study is determining the influence of social media behaviors and interactions on mental health symptoms, our main predictors comprise a variety of individual characteristics, behaviors, and interactions that users may have while using social media, including *passive and active social media use* [60], *motivations for using social media* [29], *negative social media experiences* (measured using a 4-item scale [25]), *upward social comparison on social media* (measured using a 2-item scale [61]), *cybervictimization on social media* (measured using an 8-item scale [62]), and *time elapsed during and frequency of social media usage* (measured using items previously developed by our team [63-65]).

**Demographic variables**

- We used the following items to determine study eligibility: *current location* (in or outside the United States); *age in years*(<18, 18-24, 24-29, and >30); *current sexual orientation*, measured using the item “Which of the following best describes your current sexual orientation?” (lesbian; gay; bisexual; pansexual; asexual; queer; same gender–loving; straight or heterosexual; and other, with a text box to type in) as recommended by the Sexual Minority Assessment Research Team group [57,66]; *sex assigned at birth*, measured using the question “What gender were you assigned at birth, on your original birth certificate?” (male or female); and *current gender identity*, measured using the item “Which best describes your current gender identity?” (woman, man, transgender woman, transgender man, and nonbinary or genderqueer) [57,67]. Both the current gender identity and current sexual orientation questions were also part of the attention-control questions and were asked twice (first in the eligibility screener and then in the demographics section). Other sexual identity questions included *sexual behavior*, measured using the item “In the last 5 years, who did you have sex with? By sex we mean any activity you personally define as sexual activity. Please mark all that apply” (*women, nontransgender*; *men, nontransgender*; *transgender women*; *transgender men*; *nonbinary/genderqueer*; and I have not had sex with anyone in the last 5 years) [57,66]; *sexual attraction*, measured using the question “Please indicate how sexually attracted you are to the following types of people” (*women, nontransgender*; *men, nontransgender*; *transgender women*; *transgender men*; and *nonbinary/genderqueer*) [68]; and *gender nonconformity*, measured using a 2-item scale that asked “A person’s appearance, style, or dress may affect the way people think of them. On average, how do you think people would describe your appearance, style, or dress?” and “A person’s mannerisms (such as the way they walk or talk) may affect the way people think of them. On average, how do you think people would describe your mannerisms?” Both items had responses on a 7-point Likert-type scale from “very feminine” to “very masculine” [69]. Other demographic variables included *current relationship status* (member of an unmarried couple, in a polyamorous relationship with more than 1 person, legally married or recognized civil union, and not currently in a relationship or a special commitment to someone) [57]; *current living situation*, which allowed for multiple choices (by myself; with a parent or guardian, significant other, spouse, child or children, friends, or roommate or acquaintance; or other, with a text box to type); *month and year of birth* (our team decided not to collect day of birth to preserve the privacy of study participants, crucial given the longitudinal nature of the study and the repeated contact with participants via email or SMS text messages); *race/ethnicity*, which allowed for multiple answers (American Indian or Alaskan Native; Asian or Asian American; Black or African American; Hispanic or Latina, Latino, or Latinx; Native Hawaiian or other Pacific Islander; White; or other, with a text box to type); *educational attainment* (never attended school or only attended kindergarten, grades 1 to 8, grades 9 to 11, grade 12 or general educational development, 1 to 3 years of college, college graduate, and graduate school [some or completed]); *household income* (US <$15,000, US $15,000 to <$25,000, US $25,000 to <$50,000, US $50,000 to <$75,000, US $75,000 to <$100,000, and US ≥$100,000); *current employment situation* (employed for wages; self-employed; out of work for ≥1 year; out of work for <1 year; homemaker; student; unable to work; and other, with a text box to type); *current zip code*; and *current state of residence*. Finally, the T5 survey included 1 item asking participants whether they would be willing to be recontacted within the subsequent three months for additional research opportunities *(yes or no).*

**Covariates**

- Covariates (protective and risk factors) were selected as they related to constructs included in minority stress [5], and whenever possible, we included modified items or measures to explore the same factors on social media. These included *outness in offline life*, measured using the question “In your everyday life (NOT on social media), are you out as an LGBTQ+ person to all, most, some, or to none of you’re a) family, b) straight friends c) co-workers” and the options “all, most, some, none, do not know” [57,70]; *outness on social media*, measured using a modified item that asked “On the social media site/app you use the most to keep in touch with the people you care about, are you out as an LGBTQ+ person to all, most, some, or to none of your...” with the same categories of people and options as in the previous question; *emotional and informational support offline*, measured using the PROMIS 8-item scale for these constructs [71]; *emotional and informational support on social media*, measured using a modified version of the PROMIS 8-item scale [72]; *social connectedness offline*, measured using a 20-item scale [73]; *social connectedness on social media*, measured using a 20-item scale [74]; *adverse childhood experiences*, measured using the Adverse Childhood Experiences 10-item questionnaire [75]; *loneliness*, measured using the 3-item University of California, Los Angeles Loneliness Scale [76]; *social isolation*, measured using a PROMIS 4-item scale [77]; *problematic social media use*, measured using a 6-item scale [78]; and *minority stress experiences offline and on social media*, measured using 24 items modified from the Sexual Minority Adolescent Stress Inventory (SMASI) [8]. Although the original instrument includes 11 subscales to cover a wide array of discriminatory experiences, we selected items to cover experiences that would most likely match those of young adults both offline and on social media. These included the following SMASI subscales (and their respective items): negative expectancies (3 items), negative disclosure experiences (5 items), homonegative communications (5 items), intersectionality experiences (3 items), and social marginalization (8 items).

**Additional measures**

- Although not the principal focus of our study, later waves of data collection included additional scales to explore emerging topics in the literature related to objective measures of social media use and use of geospatial dating apps. These included instructions for participants to take screenshots of their screen time app, average use time, most used apps, number of check-ins, use of mobile dating apps in the past year and past 2 weeks, number of check-ins on these apps, time elapsed during use, motivation for using dating apps, success in using dating apps, belongingness scale, perceived thriving, and reasons for stopping the use of mobile dating apps.

#### Second Series of Web-Based Interviews (T6)

The primary goal of the second qualitative phase in the PRIDE iM study is to help describe, compare, and explain the quantitative results from the longitudinal survey conducted in T2 to T5. We will group survey participants according to their outcome trajectories (eg, severe or mild depressive symptoms). Next, we will use a purposive sampling approach [38] among outcome trajectory groups identified in T2 to T5 for interviewing select “case” participants and contextualizing trajectories found in survey analyses. For example, if we identify 2 trajectory groups—“severe” and “mild” depressive symptoms—we will recruit 20 participants from each group (n=40). This approach is appropriate when the study goals include explaining significant results, positive performing cases, outliers, or confusing results [79].

The “cases” will be identified once we complete the statistical analysis of the longitudinal relationship between social media experience and behaviors and depressive symptoms. Within 30 days of completing the T5 survey, a research assistant with access to the contact information of survey participants will link the participant ID number with the respective contact information. This team member will contact “case” survey participants via email or SMS text message and invite them to participate in a web-based interview with the purpose of reviewing and contextualizing their survey results to shed light on what (if anything) other than their experiences and behaviors on social media might have influenced the results. Interested individuals will be offered to schedule a date and time for the web-based interview. Preinterview reminder activities on the day of the interview, data collection procedures and software, and postinterview transcribing will mimic those already described for T1. We plan to start recruitment for T6 in November 2022.

### Data Analysis Plan

#### Qualitative Analyses

Transcriptions will be entered into NVivo (version 12; QSR International) [80] for analysis. For both T1 and T6, we will analyze the data using reflexive thematic analysis with an experiential and realist framework [81-83]. This approach will allow us to describe and connect themes driven by the data collected from SGM participants, highlighting the specific meanings and perspectives from their lived experiences. Coding will be conducted using a hybrid inductive and deductive approach, which is appropriate given that our coding framework will partly derive from research on social media use and mental health among some groups of SGM young adults [25]. However, the paucity of research related to the impact of specific social media interactions and behaviors on depression among SGM persons requires describing concepts that are not clearly articulated in the empirical evidence available [84]. We will train independent coders and triangulate among the members of the study team to enhance trustworthiness [84].

#### Quantitative Analyses

We propose analyzing T2 to T5 data using latent growth models (LGMs) with a structural equation modeling (SEM) framework. LGMs in SEM are appropriate for our data as our assessments will measure the same construct at each data point, will have the same metric across time, and are tested at the same time intervals [85]. Crucial to our study, SEM allows for the use of latent variables or constructs in the models (eg, attitudes toward and beliefs about social media) as well as modeling mediating and moderating effects [86]. We will conduct descriptive analyses, including means, SDs, and bivariable associations between all variables included in the study. We will build SEM models at baseline and cross-sectionally and then, with each wave of longitudinal data, test whether baseline predictor characteristics are associated with depressive symptoms over the study period, as well as between- and within-person effects [87]. We will examine model fit, including fit statistics such as standardized root mean square residual and comparative fit index, according to standard guidelines [88,89]. We will use Stata software (StataCorp) [90] for conducting these analyses and follow best-practice guidelines for reporting latent trajectory studies [91].

To estimate the sample size, guidelines for estimating power to detect mediated effects using the product of coefficients test suggest that, for power >0.8 with an α level of .05, a sample size of 100 is needed to detect medium effects, and a sample size of 500 is needed to detect small effects [92]. Thus, we proposed starting at T2 with 1000 participants and, with an overall attrition rate of 50% throughout T3, T4, and T5, we will have 500 participants who completed T2 to T5, which gives us power to detect small, mediated effects. For the longitudinal mediation using LGM, a sample size of approximately 200 is needed for power >0.8 to detect a medium effect size with 5 measurement occasions. As statistical power increases as the number of measurement occasions increases and we will have 4 measurement occasions, we expect adequate power for longitudinal mediation models [92,93]. Although an attrition of 50% is expected, previous studies on risk or protective factors and health outcomes have shown that this phenomenon did not significantly affect estimates of associations between variables or demographic differences between groups [94-96].

#### Integrative Analyses

Our mixed methods study integrates quantitative and qualitative strains of data at the design, method, and interpretation and reporting levels [34,97]. We addressed the integration at the design level in the previous sections. In this section, we will describe other levels of mixed methods data integration and analyses that we will conduct in PRIDE iM. We will achieve method integration through *building* and *connecting* [97].

First, qualitative data from T1 helped us *build* the T2 to T5 quantitative surveys in that we were able to identify important constructs and potential items that were included in the questionnaires. Next, we will *connect* the quantitative data collected during T2 to T5 with the qualitative data that will be collected in T6 through purposive sampling. To do this, we will use outcome trajectories (eg, depressive symptoms) found in structural equation models and form groups based on these trajectories for inviting potential participants to the web-based interviews in T6, with the goal of describing similarities and differences across groups and explaining these differences [79].

Finally, we will integrate our quantitative and qualitative data at the interpretation and reporting level using narrative manuscripts with a *staged approach* comprising separate manuscripts to report the results of each phase of the study. This is often the case with mixed methods studies such as PRIDE iM that have a multistage approach [34]. We will use *joint displays* to bring the data together and present new insights beyond those gained from the separate quantitative and qualitative results. At all stages, we will assess the fit of data integration as recommended by the principles of best practice for mixed methods research in psychology [98,99].

## Results

Data collection will be completed by December 2022. Qualitative data analyses will be completed by March 2023, and quantitative analyses of the primary outcome of interest will be completed by June 2023.

## Discussion

### Principal Considerations

To our knowledge, PRIDE iM is the first national mixed methods study with a longitudinal quantitative phase (T2-T5) conducted in the United States to determine the relationship between a variety of social media interactions and behaviors and depression and other mental health problems that overburden SGM young adults. Moreover, our study is the first to adapt the minority stress model to explain the path by which social media interactions and behaviors may affect depressive symptoms among SGM young adults. Since the time when minority stress theory was initially developed [5], social media has disrupted most societal processes, from the way young adults make friends to the way they keep themselves informed and become involved with their communities, and this seems to be even more true for minoritized groups. The proposed study is appropriate, timely, and innovative in several aspects, from the social media–based recruitment (which allows for the participation of SGM persons who may not interface with offline recruitment venues and, thus, may be less likely to participate in research) to the study and survey design. Our study will dramatically expand on previous research [26] that focused on identifying that social media and other communication technologies influence resilience and coping among SGM youth. PRIDE iM results will provide important information about the impact of social media on mental health for researchers that seek to better understand health inequities among persons who are minoritized because of their sexual orientation, gender identity, and race or ethnicity and reduce those inequities via interventions.

### Limitations

This study has limitations to consider. First, our recruitment through social media (ie, Instagram and Facebook) might represent a barrier to participation for SGM young adults who do not use these specific platforms. However, this is unlikely given that a large majority of young adults who use social media have an Instagram or Facebook account [15]. Second, our surveys assessed social media interactions and behaviors via self-report, which subjects these reports to recall bias related to experiences on social media. To reduce this, surveys were collected every 6 to 8 weeks, and all measures asked participants only about their social media experiences “over the last 30 days.” Nevertheless, future studies would benefit from more objective methods for collecting social media data related to individuals’ interactions and behaviors. Third, we used survey assessments that are quite comprehensive, which might discourage some participants from responding to all survey items or cause them to do so hastily. To remedy this, we implemented several forms of participant engagement (eg, progressively increasing incentives, building a community around the study, and survey reminders) and data quality checks (eg, survey completion time, attention-control questions, and survey completion progress).

### Conclusions

PRIDE iM builds upon minority stress theory, a foundational model used to understand mental health disparities among minoritized groups, and will contribute to our current understanding of how sociocultural factors influence SGM individuals’ depression outcomes in a new media environment such as social media. Our study will confirm, reject, or uncover the presence of potential relationships between social media interactions and behaviors and depressive symptoms as well as temporality and direction. We will disseminate our findings via relevant academic journals and infographics on the PRIDE iM social media presence on Instagram, Twitter, and Facebook. This study will provide fundamental groundwork to develop social media–based interventions that target modifiable interactions and behaviors that are most likely to influence mental health outcomes, thus seizing the opportunity to merge the popularity of this medium among SGM people with an evidence-based approach.

## Appendix

Multimedia Appendix 1 External peer review from the National Institute on Minority Health and Health Disparities Special Emphasis Panel NIH Pathway to Independence Award (grant R00 - MD012813).

## Abbreviations

LGBTQ+: lesbian, gay, bisexual, transgender, and queer
LGM: latent growth model
SEM: structural equation modeling
SGM: sexual and gender minority

## Data Availability

The data sets generated and analyzed during this study are not publicly available yet because of (1) the ongoing nature of the study and (2) preliminary and main outcome analyses being conducted at the time of publication of this protocol, but specific data sets might be available from the corresponding author upon reasonable request.
