# Supplementary material for: The Influence of Social Media Interactions and Behaviors on Depressive Symptoms Among Sexual and Gender Minority Young Adults in the United States: Protocol for a Mixed Methods Longitudinal Study
Source: JMIR Res Protoc. 2023 Jan 24;12:e43627. doi: 10.2196/43627 (PMC9906309; doi:10.2196/43627)
Supplement: Multimedia Appendix 1 [file resprot_v12i1e43627_app1.docx]

**PROGRAM CONTACT:**

**DeLoris Hunter 301-402-1366**

[**hunterd2@mail.nih.gov**](mailto:hunterd2@mail.nih.gov)

**SUMMARY STATEMENT**

**( Privileged Communication )**

***Release Date:* 03/21/2018**

***Revised Date:***

**Principal Investigator**

**ESCOBAR-VIERA, CESAR GABRIEL**

***Application Number:* 1 K99 MD012813-01**

**Applicant Organization: UNIVERSITY OF PITTSBURGH AT PITTSBURGH**

***Review Group:* ZMD1 XLN (M1)**

**National Institute on Minority Health and Health Disparities Special Emphasis Panel NIH Pathway to Independence Award (Parent K99/R00)**

***Meeting Date:* 03/01/2018 *RFA/PA:* PA16-193**

***Council:* MAY 2018 *PCC:* IBB02DH**

***Requested Start:* 07/01/2018**

***Dual IC(s):* MH**

***Project Title:* Social Media Use and Depression Outcomes among U.S. Lesbian, Gay, and Bisexual Young Adults**

***SRG Action:* Impact Score:**

***Next Steps:* Visit https://grants.nih.gov/grants/next_steps.htm Human Subjects: 48-At time of award, restrictions will apply**

**Animal Subjects: 10-No live vertebrate animals involved for competing appl.**

**Gender: Minority: Children:**

**1A-Both genders, scientifically acceptable**

**1A-Minorities and non-minorities, scientifically acceptable 3A-No children included, scientifically acceptable**

|  |  |  |
| --- | --- | --- |
|  |  |  |
|  |  |  |
|  |  |  |
|  |  |  |

**ADMINISTRATIVE BUDGET NOTE: The budget shown is the requested budget and has not been adjusted to reflect any recommendations made by reviewers. If an award is planned, the costs will be calculated by Institute grants management staff based on the recommendations outlined below in the COMMITTEE BUDGET RECOMMENDATIONS section.**

## 1K99MD012813-01 Escobar-Viera, Cesar

**PROTECTION OF HUMAN SUBJECTS UNACCEPTABLE**

**RESUME AND SUMMARY OF DISCUSSION:** This application is submitted in response to PA16-193, NIH Pathway to Independence Award (Parent K99/R00). The purpose of this project is to determine the role of social media on depression among lesbian, gay, and bisexual young adults (LGBYA). The application has the potential to leverage evidence-based social media (SM) interventions to reduce mental health disparities for this group. This is an innovative proposal from a postdoctoral fellow who has identified an important area of training and research, related to social media usage and mental health in young adults. Because of the strength of the mentor team, there is less concern with proposed training activities. The research plan is strong and is related to a theoretical model attempting to explain the relationship of minority status, social media use, and depression. However, there are some minor concerns around the candidate's productivity and training plan. There are also concerns regarding the proposed research approach and in particular the feasibility of completing Aim 1, given the lack of statistical power with the proposed dataset to complete the desired analysis as acknowledged by the candidate. The proposal to use focus groups, stratified by sexual identity, is unclear for ability to obtain the quality of data to propose a robust modification of the minority stress model. Furthermore, there is a lack of detail on the description of methodology of sampling for Aim 3. Overall, the candidate shows promise for an independent research career given the strengths that far outweigh the weaknesses. The application is rated in the very good to excellent range and could have high impact for the affected population.

**DESCRIPTION (provided by applicant):** The overarching goal of this Pathway to Independence Award is to facilitate the development of César G. Escobar-Viera, PhD, MD, MPH, into an independent researcher, with expertise in advanced quantitative and qualitative methods to determine the role of social media on depression among lesbian, gay, and bisexual U.S. young adults (LGBYA). Ultimately, the PI seeks to leverage evidence-based SM interventions to ameliorate depression among LGBYA, which will ultimately help to reduce mental health disparities for this group. This long-term goal will be achieved via a 5-year training and research plan that will launch Dr. Escobar-Viera’s independent program of research and academic career. The career objectives of this award are to: (1) become an expert in the field of social media research applied to depression; (2) advance the PI’s knowledge in LGBT mental health disparities; and (3) gain expertise in the application of advanced quantitative and qualitative methodologies. These career objectives will be achieved via formal coursework, trainings, seminars, national conferences, mentorship, and research experience. The knowledge derived from this training plan will prepare Dr. Escobar-Viera to pursue the following aims. In the first aim, pre- existing survey data from a nationally- representative sample of young adults will be leveraged to explore cross-sectional associations between SMU and depression among LGBYA. During the second aim, qualitative assessments about SMU and depression among LGBYA. The influence of SMU on depression will be explored by inquiring about what type of use is helpful and generates social support and which use result in negative feelings and experiences (e.g., discrimination). Finally, the third aim will consist of a longitudinal examination of the associations between SMU and depression among LGBYA. Results of this aim will inform the PI’s next step of developing an SM-based intervention for LGBYA at risk for depression, mitigating identified risk factors while enhancing protective ones.

Feasibility and efficacy of the intervention will be then tested via a series of R21 and R01 projects during Dr. Escobar-Viera’s independent research career. An outstanding team of experts in LGBT research, qualitative research, longitudinal analyses, survey research, and mobile and social media interventions will provide mentorship to ensure the success of this project. Brian Primack, MD, PhD, will serve as the primary mentor. Dr. Primack leveraged his own K07 into multiple R01 and R21 awards.

Co-mentorship will be provided by Michael Marshal, PhD and Ron Stall, PhD. Moreover, Galen Switzer, PhD and Sherry Pagoto, PhD will serve as consultants. This study proposes to lay a theoretical foundation needed to develop evidence-based social media interventions to improve mental health

outcomes among sexual minorities. In addition, it will contribute to developing a model to study the influence of social media on health disparities among other minority groups. This award will forge the PI’s pathway to independence by laying a foundation for a career in social media interventions to reduce depression among LGBYA, thus decreasing health disparities for this population.

## PUBLIC HEALTH RELEVANCE

Compared with their heterosexual counterparts, lesbian, gay, and bisexual young adults (LGBYA) consistently experience higher rates of depression, which is the leading cause of disability worldwide, with a burden of $200 billion per year in the U.S. alone. Given that it is now part of the fabric LGBYA use to build and maintain social networks, it is crucial to grasp the impact SM might have on mental health, in this population. The proposed research will use an innovative version of the Minority Stress Model to understand the extent and under which circumstances characteristics of social media use might become either a risk or protective factor for depression among LGBYA.

**CRITIQUES:** The critiques below were prepared by the reviewers assigned to this application. These commentaries do not necessarily reflect the position of the authors at the close of the group discussion, nor the final majority opinion of the group, although reviewers are asked to amend their critiques if their position changed during the discussion. The resume and summary of discussion, together with the sections at the end of the summary statement, which summarize the committee’s final opinion on the use of human subjects, the inclusion of women, minorities, and children, the use of vertebrate animals, and budget are the authoritative representation of the final outcome of group discussion. If there is any discrepancy between the peer reviewers' commentaries and the numerical score on the face page of this summary statement, the numerical score should be considered the most accurate representation of the final outcome of the group discussion.

## CRITIQUE 1

Candidate: 4

Career Development Plan/Career Goals /Plan to Provide Mentoring: 4 Research Plan: 5

Mentor(s), Co-Mentor(s), Consultant(s), Collaborator(s): 1 Environment Commitment to the Candidate: 1

## Overall Impact:

The candidate for this K99/R00 will have completed 3-years of postdoctoral training, two of them at the institution where he proposes to conduct the K99 training. His proposal is strong in that he has identified an important an innovative area of training and research, related to social media usage in young adults, and addressing an important public health concern in focusing on lesbian, gay, and bisexual people and depressive symptoms. There are some weaknesses in the application, including that the training activities are vague—they comprise of taking some courses and working with mentors—and therefore raise questions about how they are distinct from the candidate’s post-doctoral training experience with the same mentors. The research plan is stronger, in that it is related to a theoretical model attempting to explain the relationship of minority status, social media use, and depression. The plan to use a probability sample of lesbians, gay men, and bisexuals aged 18 – 30 is also a strength. But the research plan lacks in detail, for example, there is not much information on the substantive areas of inquiry in the qualitative research, and little description of the sampling approach and feasibility of assembling a sample of 3,500 young lesbians, gay men, and bisexuals (and for what appears like a rather low cost). This reviewer is also unclear about how the research plan to study the impact of social media use connects with the broader goal of informing interventions that leverage social media to prevent depression.

## Candidate:

**Strengths**

- - The candidate has made strong connections with an excellent team of researchers who support his goals and are dedicated to help in his mentorship.

## Weaknesses

- - The candidate is completing post-doctoral training with the same faculty and at the same institution and on the same topics as his proposed continued training. It is not clear how the post-doctoral training relates to his K99 training or why additional training is needed.
  - The candidate’s productivity has been moderate, with only 3 publications to date and it seems, based on his biosketch, with 2 first authored. The letter from Mentor, Primack, suggests that more recently the candidate has been productive and is involved with 9 or 12 publications. (The biosketch includes manuscripts in preparations and others that do not have publication date, so it is difficult to assess).

## Career Development Plan/Career Goals & Objectives/Plan to Provide Mentoring: Strengths

- - The candidate’s goals are to develop as an independent researcher to study social media use and depression among lesbian, gay, bisexual young adults. This is an innovative area of research with interesting complexities, as social media use has become a prominent mode of communication with both positive and negative implications for health.

## Weaknesses

- - Career goals are broad and appropriate for a career in research, but also a bit vague in terms of how to get there. For example, they often rely on the expertise of mentors, which is impressive, but with no sufficient information about how the expertise of mentors will be imparted. For example, career goal 2 is to “Advance my knowledge in LGB mental health disparities,” with some specific goals like learn about “stress, racism, environment . . . “but the plan for training states rather vaguely “Coursework, research lab meetings, and conference attendance will supplement my training in this area.” The didactics section describes a course on LGBT research, attending lab meetings, and conferences. These seem valued, but not very specific to the goal. This reviewer is especially concerned about this lack of detail because the candidate will have already spent 2 years as a post-doctoral student at the same institution with the same mentors.

## Research Plan:

**Strengths**

- - The use of probability samples is a strong element of the research plan.
  - The development of a theoretical model by the candidate will be used to test hypotheses and provides excellent guidance to his research plan.

## Weaknesses

- - It’s not clear that the question regarding interventions using social media are answered by investigating whether social media causes depression. It seems to be quite different to ask how social media can be leveraged to intervene than to ask whether social media use is itself a cause of depression. The research plan seems to straddle both aspects of this question, with most of the efforts addressing social media as a cause of depression (or as ameliorating depression) but not specifically how to leverage social media to prevent depression.
  - The focus on disparities is also tenuous. If social media use explains disparities than it has to be differentially cause depression in heterosexual vs. LGB people, but that does not seem to be a focus of the research.
  - If Aim 1 uses an existing study, why is there no better description of the study population and the LGB respondents therein.
  - The qualitative study is poorly described, there is no indication as to what questions will be asked (an interview outline would have helped or even interview topics). Also, there is no clear justification for the use of focus groups. The reviewer can see an argument that individual interviews will lead to better disclosure of personal narratives about depression and social media use as opposed to more socially desirable answers.
  - There is no clear description of how a sample of 3,500 lesbian, gay, and bisexual individuals aged 18-30 who are representative of the U.S. population will be accomplished (the current study using Qualtrics has 2,400 adults of all sexual orientations).

## Mentor(s), Co-Mentor(s), Consultant(s), Collaborator(s): Strengths

- - The candidate has assembled an excellent group of mentors and consultants with expertise in areas relevant to the proposed career and research aims.
  - The candidate has an established relationship with the mentors and consultant and they provided strong letters of support.

## Weaknesses

- - None.

## Environment and Institutional Commitment to the Candidate: Strengths

- - The environment seems to promise successful accomplishment of the career and research goals.

## Weaknesses

- - None.

## Protections for Human Subjects:

Unacceptable Risks and/or Inadequate Protections

- - Sufficient human subjects protections as this study involves minimal risk, but greater attention needs to be paid to the invited focus group participation. Even though individuals had indicated that they are willing to participate in further study, they may not be willing to reveal themselves in a focus group with other, unknown participants. To the extent that participants have not revealed their LGB status to others, this reviewer doesn't see how their confidentiality can be assured under the current design.

Data and Safety Monitoring Plan (Applicable for Clinical Trials Only): Not Applicable (No Clinical Trials)

## Inclusion of Women, Minorities and Children:

- - Sex/Gender: Distribution justified scientifically.
  - Race/Ethnicity: Distribution justified scientifically.
  - For NIH-Defined Phase III trials, Plans for valid design and analysis: Not applicable.
  - Inclusion/Exclusion of Children under 18: Excluding ages <18; justified scientifically.

## Vertebrate Animals:

Not Applicable (No Vertebrate Animals)

## Biohazards:

Not Applicable (No Biohazards)

## Training in the Responsible Conduct of Research:

Acceptable

Comments on Format (Required):

- - Acceptable.

Comments on Subject Matter (Required):

- - Acceptable.

Comments on Faculty Participation (Required; not applicable for mid- and senior-career awards):

- - Acceptable.

Comments on Duration (Required):

- - Acceptable.

Comments on Frequency (Required):

- - Acceptable.

## Select Agents:

Not Applicable (No Select Agents)

## Resource Sharing Plans:

Not Applicable (No Relevant Resources)

## Authentication of Key Biological and/or Chemical Resources:

Not Applicable (No Relevant Resources)

## Budget and Period of Support:

Recommend as Requested

## CRITIQUE 2

Candidate: 3

Career Development Plan/Career Goals /Plan to Provide Mentoring: 2 Research Plan: 4

Mentor(s), Co-Mentor(s), Consultant(s), Collaborator(s): 1 Environment Commitment to the Candidate: 1

## Overall Impact:

The candidate is proposing to study the association between social media use and depression. Because depression among SGM youth is common and because youth in general tend to be high users of social media, this is a worthwhile line of research. There is much we do not yet know about social media’s effect on mental health. Further, the candidate is a psychiatrist with postdoctoral affiliations in the Center for LGBT Health and the Center for Research on Media, Technology, and Health, so he has the level of mentored support needed for success. Enthusiasm for this application was lessened when reviewing the candidate’s publication record and training and research plan. While the candidate has three publications in this area and more forthcoming, the overall publication productivity for someone in a second postdoc is somewhat lower than expected, and the training plan lacks detail. The first aim is underpowered and the proposed use of 6-8 focus groups rather than interviews to inform the adaptation of the minority stress model is questionable.

## Candidate:

**Strengths**

- - Candidate is a psychiatrist and outcome of interest is depression.
  - Candidate has published on social media and has a postdoc in Center for LGBT Health Research.
  - One of his mentors is Director of Center for Research on Media, Technology, and Health.

## Weaknesses

- - Low publication record.

## Career Development Plan/Career Goals & Objectives/Plan to Provide Mentoring: Strengths

- - Candidate provided a mentoring plan that seems appropriate for his study/goals.

## Weaknesses

- - More details are needed about how the training plan is different from normal postdoctoral training opportunities.

## Research Plan:

**Strengths**

- - Because of his mentor’s previous research, the candidate has access to existing datasets/population from which to recruit.
  - Proposed aims are appropriate to answer the candidate’s research question.

## Weaknesses

- - Aim 1 is underpowered, raising concerns about why this particular dataset is being proposed to answer the research question.
  - In Aim 2, the candidate is proposing 6-8 focus groups, “stratified by specific sexual minority group… [to gain] an in-depth understanding of motivations [and] useful SMU and negative experiences on social media” in order to modify a proposed minority stress model. This reviewer has concerns about the decision to use focus groups to gain an in-depth understanding of motivations and negative experiences; in-depth interviews might be more appropriate. This reviewer also has concerns about the number of focus groups. Presumably, stratification is being proposed because the candidate assumes the motivations and experiences differ between lesbians, gay men, and bisexual persons (it is unclear if the candidate is proposing to stratify on bi-men and bi-women or treat all bisexual persons the same). If there are a maximum of 8 focus groups and four strata, then, only 2 focus groups are being proposed per strata, so the candidate is unlikely to reach saturation, which would seem necessary to make an informed adaptation of the minority stress model.
  - Aim 3 seem appropriate.

## Mentor(s), Co-Mentor(s), Consultant(s), Collaborator(s): Strengths

- - The candidate has an outstanding team of mentors.

## Weaknesses

- - None.

## Environment and Institutional Commitment to the Candidate: Strengths

- - The candidate is affiliated with the labs needed to complete his project.

## Weaknesses

- - None.

## Protections for Human Subjects:

Acceptable Risks and Adequate Protections

- - The discussion of human subjects risks and benefits is appropriate. Data and Safety Monitoring Plan (Applicable for Clinical Trials Only):

Not Applicable (No Clinical Trials)

## Inclusion of Women, Minorities and Children:

- - Sex/Gender: Distribution justified scientifically.
  - Race/Ethnicity: Distribution justified scientifically.
  - For NIH-Defined Phase III trials, Plans for valid design and analysis: Not applicable.
  - Inclusion/Exclusion of Children under 18: Excluding ages <18; justified scientifically.
  - Inclusion criteria are appropriate for the proposed study.

## Vertebrate Animals:

Not Applicable (No Vertebrate Animals)

## Biohazards:

Not Applicable (No Biohazards)

## Training in the Responsible Conduct of Research:

Acceptable

Comments on Format (Required):

- - CITI and Pitt workshops. Comments on Subject Matter (Required):
  - Appropriate.

Comments on Faculty Participation (Required; not applicable for mid- and senior-career awards): Comments on Duration (Required):

- - Appropriate.

Comments on Frequency (Required):

- - Appropriate.

## Select Agents:

Not Applicable (No Select Agents)

## Resource Sharing Plans:

Not Applicable (No Relevant Resources)

## Authentication of Key Biological and/or Chemical Resources:

Not Applicable (No Relevant Resources)

## Budget and Period of Support:

Recommend as Requested

## CRITIQUE 3

Candidate: 3

Career Development Plan/Career Goals /Plan to Provide Mentoring: 2 Research Plan: 3

Mentor(s), Co-Mentor(s), Consultant(s), Collaborator(s): 2 Environment Commitment to the Candidate: 2

## Overall Impact:

The candidate proposes to examine the role of social media and depression among lesbian, gay, and bisexual young adults. His primary mentor leveraged his own K award for successful R awards.

Proposes a combination of mentoring, didactic, and research experiences to meet career development

objectives. The research plan lacks details specific to qualitative methods and sample acquisition feasibility.

## Candidate:

**Strengths**

- - Trained in health technology with research experience in social media and depression in lesbian, gay, bisexual populations.
  - Some publications in these areas.

## Weaknesses

- - Small number of published manuscripts.
  - Lack of distinction between post-doc training and K99 career development training.

## Career Development Plan/Career Goals & Objectives/Plan to Provide Mentoring: Strengths

- - Combination of mentoring, didactic, and research activities.

## Weaknesses

- - Lack of details related to specific contributions of mentors and how courses will contribute to overall career development.

## Research Plan:

**Strengths**

- - Research strategic includes a strong scientific premise; if successful, is likely to have significant impact.

## Weaknesses

- - Limited innovation, a minor flaw in a training award.
  - Tenuous focus on disparities.
  - Poor description of the qualitative study; no interview guide, draft questions, or topic guide.
  - Lack of details related to sample acquisition.
  - Aim 1 maybe under powered.

## Mentor(s), Co-Mentor(s), Consultant(s), Collaborator(s): Strengths

- - The scientific expertise of the mentor and co-mentors are complimentary and appropriate.

## Weaknesses

- - None noted.

## Environment and Institutional Commitment to the Candidate: Strengths

- - The institutional commitment appears adequate, as documents in letters of support.

## Weaknesses

- - None noted.

## Protections for Human Subjects:

Acceptable Risks and Adequate Protections

Data and Safety Monitoring Plan (Applicable for Clinical Trials Only): Not Applicable (No Clinical Trials)

## Inclusion of Women, Minorities and Children:

- - Sex/Gender: Distribution justified scientifically.
  - Race/Ethnicity: Distribution justified scientifically.
  - For NIH-Defined Phase III trials, Plans for valid design and analysis: Not applicable.
  - Inclusion/Exclusion of Children under 18: Excluding ages <18; justified scientifically.

## Vertebrate Animals:

Not Applicable (No Vertebrate Animals)

## Biohazards:

Not Applicable (No Biohazards)

## Training in the Responsible Conduct of Research:

Acceptable

Comments on Format (Required):

- - Adequate.

Comments on Subject Matter (Required):

- - Adequate.

Comments on Faculty Participation (Required; not applicable for mid- and senior-career awards):

- - Adequate.

Comments on Duration (Required):

- - Adequate.

Comments on Frequency (Required):

- - Adequate.

## Select Agents:

Not Applicable (No Select Agents)

## Resource Sharing Plans:

Acceptable

## Authentication of Key Biological and/or Chemical Resources:

**Budget and Period of Support:**

Recommend as Requested

## THE FOLLOWING SECTIONS WERE PREPARED BY THE SCIENTIFIC REVIEW OFFICER TO SUMMARIZE THE OUTCOME OF DISCUSSIONS OF THE REVIEW COMMITTEE, OR REVIEWERS’ WRITTEN CRITIQUES, ON THE FOLLOWING ISSUES:

**PROTECTION OF HUMAN SUBJECTS: UNACCEPTABLE**

During the discussion, it’s noted that there was a lack of details on how the confidentiality of the participants in the invited focus group can be assured under the current design.

## INCLUSION OF WOMEN PLAN: ACCEPTABLE INCLUSION OF MINORITIES PLAN: ACCEPTABLE INCLUSION OF CHILDREN PLAN: ACCEPTABLE

**COMMITTEE BUDGET RECOMMENDATIONS: The budget was recommended as requested.**

Footnotes for 1 K99 MD012813-01; PI Name: Escobar-Viera, Cesar Gabriel

NIH has modified its policy regarding the receipt of resubmissions (amended applications). See Guide Notice NOT-OD-14-074 at <http://grants.nih.gov/grants/guide/notice-files/NOT-OD-> 14-074.html. The impact/priority score is calculated after discussion of an application by averaging the overall scores (1-9) given by all voting reviewers on the committee and multiplying by 10. The criterion scores are submitted prior to the meeting by the individual reviewers assigned to an application, and are not discussed specifically at the review meeting or calculated into the overall impact score. Some applications also receive a percentile ranking. For details on the review process, see [http://grants.nih.gov/grants/peer_review_process.htm#scoring.](http://grants.nih.gov/grants/peer_review_process.htm#scoring)

**MEETING ROSTER**

The roster for this review meeting is displayed as an aggregated roster that includes reviewers from multiple MD Special Emphasis Panels of the Aggregate Roster for NIMHD Special Emphasis Panels for the 2018/05 council round.

This roster for MD is available at: [http://public.era.nih.gov/pubroster/Reports?DOCTYPE=SEP&DESFORMAT=PDF&AGENDA_SEQ_NUM](http://public.era.nih.gov/pubroster/Reports?DOCTYPE=SEP&amp;DESFORMAT=PDF&amp;AGENDA_SEQ_NUM_P=338507)

[_P=338507](http://public.era.nih.gov/pubroster/Reports?DOCTYPE=SEP&amp;DESFORMAT=PDF&amp;AGENDA_SEQ_NUM_P=338507)
